# High-Quality Draft Genome Sequence of *Francisella tularensis* subsp. *holarctica* Strain 08T0073 Isolated from a Wild European Hare

**DOI:** 10.1128/genomeA.01577-16

**Published:** 2017-03-23

**Authors:** Anne Busch, Prasad Thomas, Kerstin Myrtennäs, Mats Forsman, Silke Braune, Martin Runge, Herbert Tomaso

**Affiliations:** a Friedrich-Loeffler-Institut, Institute of Bacterial Infections and Zoonoses, Jena, Germany; b Division of CBRN Security and Defence, Swedish Defence Research Agency (FOI), Umeå, Sweden; c Food and Veterinary Institute, Lower Saxony State Office for Consumer Protection and Food Safety, Hannover, Germany

## Abstract

Here, we report a high-quality draft genome sequence of *Francisella tularensis* subsp. *holarctica* strain 08T0073, isolated from the cadaver of a wild European hare (*Lepus europaeus*) found near Helmstedt, Lower Saxony, Germany, in 2007. In Germany, infected hares are a major source of tularemia in humans.

## GENOME ANNOUNCEMENT

*Francisella tularensis* is a Gram-negative bacterial pathogen and the causative agent of tularemia. *F. tularensis* subsp. *holarctica* seems to be a reemerging pathogen in Germany infecting many animal species. In Germany, the European hare (*Lepus europaeus*) is considered the most common source of infection in humans (1–3).

Here, we describe the genome sequence of *F. tularensis* subsp. *holarctica* strain 08T0073 (also known as FDC386 and W2990), which caused fatal tularemia in a hare. Cultivation of bacteria from organ specimens was successful on cysteine heart agar at 37°C with 5% CO_2_ for 48 h. Antibiotic resistance showed resistance to erythromycin. The phylogeny of the strain was further elucidated using real-time PCR assays and was characterized as genetic clade B.12, subclade B.33/34/75. Analysis with CanSNPer (https://github.com/adrlar/CanSNPer) revealed a new subclade designated B.80. The new canonical single-nucleotide polymorphism B.80 at position 769711 in FSC200 (GenBank accession no. CP003862.1) was selected to define the new subclade and was deposited.

DNA for whole-genome sequencing was prepared from a 10-ml culture in brain heart infusion broth. Bacterial cells were harvested after 72 h by centrifugation, and the DNA was purified using QIAGEN Genomic-tip 20/G and a QIAGEN Genomic DNA buffer set kit (Qiagen, Hilden, Germany). The sequencing library was generated using the Nextera XT DNA library prep kit (Illumina, Inc., San Diego, CA, USA). From an Illumina MiSeq run with an average read length of 250 bp and an expected insert size of 350 bp, 1.5 million paired-end reads were generated (mean sequencing depth of >160 reads). Further read processing included quality-trimming (with parameters set to 0.01 as quality score and two as the maximum number of ambiguities permitted), merging paired reads, and *de novo* assembly with read-mapping (CLC Genomics Workbench version 8.5.1). Annotation was performed with the RAST server and Prokka with the recommended standard settings (4, 5). The RAST genome assembly was represented by 97 contigs with an *N*_50_ contig length of 27,460 bp. The final assembled genome consisted of 1,775,487 bp with a G+C content of 32.2%. Annotation features include 1,949 genes and 38 RNAs. The annotated content of four rRNAs showed a higher coverage than the genome coverage and hence is underrepresented compared to 10 rRNAs found in the complete genomes NC_007880 (LVS) and NC_008369 (OSU18). This is probably due to the low resolution of short reads to resolve multicopy rRNA genes during assembly. Analyses based on sequence identity with RAST revealed the closest relationships to the genomes of *F. tularensis* subsp. *holarctica* LVS (live vaccine strain) and *F. tularensis* subsp. *holarctica* strain FSC257. The closest related public genome identified by genome alignment and phylogenetic analysis was A271_1 (FDC408, sample accession no. SAMN03773882), isolated from a beaver in Berlin (subclade B.75). Consistently, the draft genome contained the *Francisella* pathogenicity island, missing the genes coding for the hypothetical proteins PdpD1 und PdpD2 (6).

### Accession number(s).

This whole-genome shotgun project has been deposited in DDBJ/ENA/GenBank under the accession number MRZT00000000. The version described in this paper is the first version, MRZT01000000.
